# Childhood Adversities and Physical and Mental Health Outcomes in Adults Living with HIV: Findings from the Ontario HIV Treatment Network Cohort Study

**DOI:** 10.1155/2018/2187232

**Published:** 2018-03-01

**Authors:** Tsegaye Bekele, Evan J. Collins, Robert G. Maunder, Sandra Gardner, Sergio Rueda, Jason Globerman, Thao Lan Le, Jon Hunter, Anita Benoit, Sean B. Rourke, The OHTN Cohort Study Team

**Affiliations:** ^1^The Ontario HIV Treatment Network, Toronto, ON, Canada; ^2^Immunodeficiency Clinic, Toronto General Hospital, Toronto, ON, Canada; ^3^Department of Psychiatry, University of Toronto, ON, Canada; ^4^Department of Psychiatry, Mount Sinai Hospital, Toronto, ON, Canada; ^5^Baycrest Health Sciences, Toronto, ON, Canada; ^6^Dalla Lana School of Public Health, University of Toronto, ON, Canada; ^7^Institute for Mental Health Policy Research, Centre for Addiction and Mental Health, Toronto, ON, Canada; ^8^Women's College Research Institute, Women's College Hospital, Toronto, ON, Canada; ^9^Centre for Urban Health Solutions, Li Ka Shing Knowledge Institute, St. Michael's Hospital, Toronto, ON, Canada; ^10^Ontario HIV Treatment Network, Toronto, ON, Canada

## Abstract

We sought to estimate the prevalence of childhood adversity and examine its relationship with health outcomes among people living with HIV. Study participants included 1409 adults living with HIV and receiving care in Toronto, Canada. Data on childhood adversity, health behaviors, HIV outcome measures, depression, and health-related quality of life (HRQOL) were collected through face-to-face interviews and medical records. Statistical analyses included multivariable linear and logistic regression modeling. The prevalence of any childhood adversity was 71% (individual types ranged from 11% to 44%) and higher prevalence was associated with younger age, Indigenous or African/Caribbean/Black ethnicity, lower socioeconomic status, and higher rates of cigarette smoking and nonmedicinal drug use. Greater number of childhood adversities was associated with greater odds of depression and decreasing mental HRQOL. HIV care providers need to screen for childhood adversities and address childhood trauma within the context of HIV care.

## 1. Introduction

Traumatic life experiences like childhood physical and sexual abuse have been associated with adverse mental and physical health outcomes in adulthood. The Adverse Childhood Experiences (ACE) study followed over 17,000 participants and showed robust associations between a wide range of traumatic experiences in childhood and increased rates of somatization, negative health behaviors (e.g., alcoholism, high number of sexual partners), adverse mental and physical health outcomes, and mortality [1]. In a more recent study among 48,526 US adults, a higher ACE score was associated with increased odds for binge and heavy drinking, cigarette smoking, HIV risk behaviors, diabetes, myocardial infarction, coronary artery disease, stroke, depression, and health disability [2]. Similar Canadian and European studies have also linked childhood adversities with negative health outcomes [3–6].

History of childhood trauma is more prevalent in people living with HIV than the general population. A recent review of HIV and trauma concludes that the prevalence of trauma is 1.5–2 times greater in people living with HIV than the general population and that both HIV infection and trauma constitute a syndemic, that is, two or more conditions that occur together, interact synergistically, and result in excess burden of disease [7]. Although retrospective rates of trauma vary widely in different populations, most studies concluded that it is disproportionately high in people living with HIV, particularly among women, ethnic minorities including Indigenous people, men who have sex with men (MSM), and drug users [8–15].

As observed in the general population, studies have shown associations between childhood trauma and negative health outcomes. A review has shown associations between trauma and poorer health outcomes both psychiatric and physical, as well as HIV medication adherence and health behaviors [16]. Notably, the above reviews include trauma in both childhood and adulthood although a systematic review specific to childhood trauma concluded that, among people living with HIV, childhood maltreatment is associated with a broad range of mental health outcomes, including substance disorders, depression, posttraumatic stress disorder, and poor adherence to ARVs [17]. In a series of studies from the CHASE cohort, for example, trauma independently and significantly predicted outcomes as diverse as poor antiretroviral (ARV) adherence, increased transmission risk behaviors, increased healthcare utilization, HIV disease progression, and higher HIV-related and all-cause mortality rates among people living with HIV [8, 18].

The present research sought to address the issue of childhood adversity and health outcomes in adulthood using data from the Ontario HIV Treatment Network Cohort Study (OCS), a large observational cohort of adults living with HIV accessing care in Ontario, Canada. Most of the research cited above focuses on childhood physical and sexual abuse, as opposed to the broader construct of childhood adversity, which includes both violent and nonviolent trauma. Our study sought to determine the prevalence of childhood adversities in adults living with HIV and to examine the relationship between childhood adversity and mental and physical health in this population, using depression and health-related quality of life (HRQOL) as primary outcomes.

## 2. Methods

### 2.1. Study Sample

The current study uses secondary data collected from 1,409 adults living with HIV and enrolled in the OCS at three sites in Toronto, Canada. The OCS is an open observational cohort of adults in HIV care in Ontario, Canada. Details of the cohort have been described elsewhere [19]. Briefly, recruitment takes place at 11 hospital-based specialty HIV clinics, hospital-based family practice units, and community-based primary care physician practices, and participants broadly represent people receiving HIV care in Ontario. Eligibility criteria included the following: (a) 16 years of age or older; (b) diagnosed with HIV infection; (c) resident of Ontario; (d) able to speak English; and (e) able to provide informed consent. Clinical data were abstracted from participants' medical records and enhanced through linkage with databases at Public Health Ontario Laboratories, which conduct all serological, viral, and bacteriological tests in the province. Demographic, behavioral, psychosocial, and HRQOL data were collected using standardized questionnaires through annual face-to-face interviews [19]. The OCS was approved by the ethics review boards of individual study sites and the University of Toronto.

### 2.2. Measures

#### 2.2.1. Childhood Adversities

Data on childhood adversities were collected using the National Population Health Survey (NPHS) stress questionnaire [20, 21], which includes subscales on acute stress, chronic stress, and childhood adversities. The childhood adversity subscale asked 7 questions “about some things that may have happened to you while you were a child or a teenager, before you moved out of the house.” The questions included the following:

(1) Did you spend 2 weeks or more in a hospital* (lengthy hospital stay)*?

(2) Did your parents get a divorce* (parental divorce)*?

(3) Did either of your parents not have a job for a long time when they wanted to be working* (lengthy parental unemployment)*?

(4) Did something happen that scared you so much that you thought about it for years after* (scary event)*?

(5) Were you sent away from home because of something you did wrong* (sent away from home)*?

(6) Did either of your parents drink or use drugs so often that it caused problems for the family* (frequent parental substance use)*?

(7) Were you ever physically abused by someone close to you* (physical abuse)*?

Information on childhood adversities was solicited at first questionnaire for the majority of participants. For others, who provided information on repeated visits, we developed an algorithm to choose the “worst case” final scenario. The internal consistency of the childhood adversities subscale in our sample was acceptable (*α* = 0.73). Positive responses to the 7 questions were summed with scores categorized as 0, 1, 2, 3, 4, and ≥5 adversities endorsed.

#### 2.2.2. Health Outcomes

*Depressive Symptoms*. Depressive symptoms during the past week were assessed using the Center for Epidemiological Studies-Depression scale (CES-D) [22]. The CES-D includes 20 items comprising six scales that reflect the major dimensions of depression: depressed mood, feelings of guilt and worthlessness, feelings of helplessness and hopelessness, psychomotor retardation, loss of appetite, and sleep disturbance. Responses indicated the frequency of occurrence of each symptom and are scored on a 4-point scale ranging from 0 (rarely or none of the time) to 3 (most or all of the time). Internal consistency of the scale in our sample was excellent (Cronbach's alpha = 0.94). A total score (possible range: 0 to 60) was computed by summing responses to all items with higher scores indicative of higher symptom burden. We used a threshold of a score of ≥16 to determine presence of clinically significant depression [22].

*Health-Related Quality of Life*. We used the standard version (4-week recall period) of the Medical Outcomes Study 36-item short-form health survey (SF-36v2) to assess physical and mental health-related quality of life [23]. The SF-36v2 is a generic health survey that has been validated with HIV-positive populations and was found to be suitable to monitor the health status [24]. It assesses eight domains of health: physical functioning, role functioning physical, bodily pain, general health, vitality, social functioning, role functioning emotional, and mental health. Internal consistencies of the eight domains were good (Cronbach's alpha ranged from 0.76 for general health domain to 0.93 for role functioning domain). Following an established guideline [25], we first computed scores for all eight domains following developers' scoring manual and then generated two composite scores, Physical Component Summary (PCS) and Mental Component Summary (MCS), from the eight subscales. The PCS and MCS scores have a possible range of 0 to 100, with higher scores indicating better physical and mental health. The minimum clinically significant differences are 2-3 points for PCS score and 3 points for MCS score [25].

#### 2.2.3. Covariates

Data on demographic and clinical covariates were extracted from the most recent questionnaire for the purpose of this analysis. Demographic variables collected included age (<30 versus 31–40 versus 41–50 versus >50), gender/sexual orientation (female versus male, MSM versus male, non-MSM), race or ethnicity (Indigenous versus African, Caribbean, or Black versus White versus other, non-White), country of birth (born in Canada versus immigrant), marital status (married or living with a partner versus single, divorced, or widowed), education (some high school or less versus completed high school versus some postsecondary versus completed university), employment status (employed versus unemployed-seeking versus not in the labour force versus on disability), and annual personal income (<$20,000 versus $20,000 to $49,999 versus ≥$50,000). Alcohol use in the past 12 months was assessed using the first 3 items of Alcohol Use Disorders Identification Test alcohol consumption questions (AUDIT-C) scale and categorized into hazardous alcohol (AUDIT-C score ≥ 4 cut-off for men and AUDIT-C ≥ 3 cut-off for women) versus nonhazardous alcohol using previously established criteria [26]. Participants were also asked about cigarette smoking in the past month (current smoker versus former smoker versus nonsmoker) and use of nonmedicinal drugs (excluding marijuana) in the past six months (yes versus no). We extracted clinical information including CD4 count (<200 cells/mm^3^ versus 200–499 cells/mm^3^ versus ≥500 cells/mm^3^), HIV viral load (<50 copies/mL versus ≥50 copies/mL), diagnosis of AIDS (yes versus no), date of HIV diagnosis, and current antiretroviral treatment (yes versus no) from medical charts. Information on date of HIV diagnosis was augmented with self-report where data were missing in medical charts. Sexual activity, partner's HIV status, and use of condoms in the past 3 months were also assessed.

### 2.3. Statistical Analysis

All statistical analyses were performed using SAS software (SAS Institute Inc., version 9.4). We calculated prevalence of childhood adversities (individual events as well as overall) as the number of individuals reporting the event divided by the total number of participants. Confidence intervals of prevalence were estimated using binomial proportion distributions. Demographic, socioeconomic, substance use and biomedical characteristics of the sample were summarized using descriptive statistics (frequencies and percentages for categorical variables). We used Kruskal Wallis one-way analysis of variance test (continuous variables) and Chi-square tests (categorical variables) to examine the associations between number of childhood adversities and demographic, substance use and clinical characteristics.

Multivariable linear and logistic regression models were fitted with health outcomes as dependent variables, and the number of childhood adversities counts as the main predictor variable. Participants with no childhood adversity were used as a referent group. Confounder variables for multivariable regression models were selected based on prior knowledge as well as bivariable analyses (*p* < 0.10). When two or more variables measured similar concepts or showed strong correlation (e.g., employment and income), we retained the variable that showed the strongest association with the outcome variable. We also checked for effect modification by adding interaction terms between number of childhood adversities and other covariates included in final regression models and reported only those that were significant at *p* < 0.05 level. All reported *p*-values are from two-tailed tests with *p* < 0.05 indicating statistical significance.

## 3. Results

Demographic and other characteristics of study participants are summarized in Table 1. The majority of the study participants were MSM (62%), over 40 years old (77%), White (55%) and were born in Canada (56%). Nearly one-third (30%) have completed university and 48% were employed full-time or part-time. Forty-two percent of the participants reported an annual income of less than $20,000 and 25% had an annual income of $50,000 or higher.

### 3.1. Prevalence of Childhood Adversity

Overall, 71.3% (95% CI: 68.9%–73.7%) of participants reported experiencing at least one childhood adversity (Table 1). Prevalence of childhood adversity was inversely associated with age; that is, higher percentage of younger participants reported childhood adversities than older participants. Although a slightly higher percentage of female participants reported one or more childhood adversity than male participants, the difference was not statistically significant. The prevalence also varied by ethnicity with highest rates among Indigenous participants and the lowest rate among “other, non-White” participants. Canadian-born participants were as likely as immigrants to report experiencing childhood adversities. Participants with lower levels of education, who were unemployed, or with lower income reported significantly higher prevalence of childhood adversity. We also found differences in prevalence of childhood adversity between smokers versus nonsmokers and people who used drugs (except marijuana) versus those who abstained from using drugs. Relative to participants with no childhood adversities, those with childhood adversity reported higher prevalence of unprotected sex with a sexual partner whose HIV status was negative or unknown. Higher prevalence of child adversities was also observed among participants who were not on ART and had detectable viral load and lower CD4 count.

The three most common types of childhood adversities reported were a* scary traumatic event* (43.9%, 95% CI: 41.3%–46.5%),* physical abuse* (27.8%, 95% CI: 25.4%–30.2%), and* frequent parental substance use* (26.3%, 95% CI: 24.1%–28.7%) (Supplemental Table 1). Younger participants reported significantly (*p* values < 0.05) higher prevalence of* parental divorce*,* lengthy parental unemployment*,* scary traumatic event*, and being* sent away from home* than older participants. Females had higher prevalence of* physical abuse* than men while male-MSM participants reported higher prevalence of* frequent parental substance use* than female or non-MSM participants. Non-MSM participants reported higher rate of being* sent away from home* than MSM. Indigenous participants reported significantly higher prevalence of* frequent parental substance use* and* physical abuse* while* scary traumatic event* was more common among African, Caribbean, or Black participants than other ethnic groups. Canadian-born individuals had significantly higher prevalence of* frequent parental substance u*se than immigrants. We also noted that among the seven childhood adverse items, physical abuse was strongly correlated (all *p* values < 0.01) with “scary traumatic event” (*r* = 0.37), “frequent parental alcohol or drug use” (*r* = 0.28), being “sent away from home” (*r* = 0.27), and “lengthy parental unemployment” (*r* = 0.19).

We also examined the number of childhood adversities in our sample (Table 2). The frequencies were as follows: 1 adversity = 350 (24.8%); 2 adversities = 259 (18.4%); 3 adversities = 163 (11.6%); 4 adversities = 135 (9.6%); and 5 or more adversities = 98 (7.0%). Greater number of adversities were significantly associated (*p* < 0.05) with younger age, lower education level, unemployment, and lower income. Although women had higher prevalence of any childhood adversity than men, number of childhood adversities did not vary significantly by gender. We found no statistically significant association between number of reported childhood adversities and ethnicity, country of birth, or marital status. Increasing number of childhood adversities was associated with substance use. Cigarette smoking rate increased linearly from 23% among those with no adversities to 41% among those who reported five or more adversities. Prevalence of nonmedicinal drugs use varied (range: from 9% to 28%) by number of adversities in a nonlinear fashion. There was no significant association between number of childhood adversities and hazardous alcohol use (*p* = 0.667). Greater number of childhood adversities was also inversely associated with being on ART and having undetectable HIV viral load but was not associated with CD4 count.

### 3.2. Childhood Adversities and Health Outcomes

**Figure 1:**
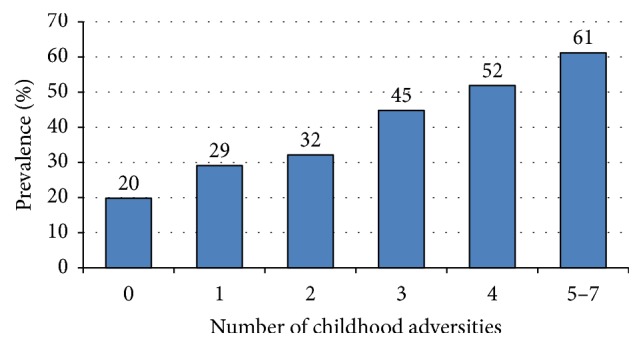
Prevalence of depression (CES-D ≥ 16) by number of childhood adversities.* Note*. Trend was significant (Chi-square trend test; *χ*^2^ = 99.0; *p* < 0.001).

**Figure 2:**
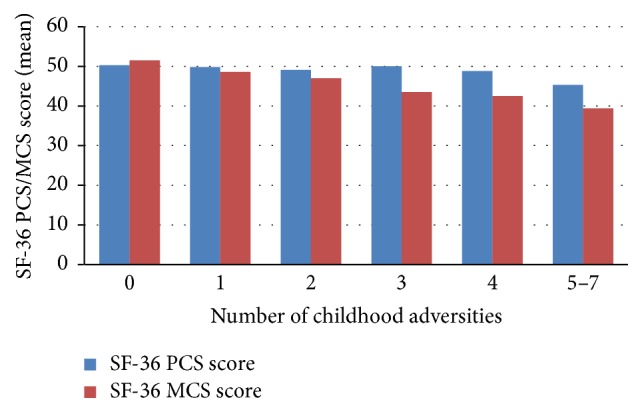
SF-36 PCS and SF-36 MCS scores by number of childhood adversities.* Note*. There was a significant linear trend for SF-36 MCS score (ANOVA trend test; *F* = 77.2.9; *p* < 0.001), but not for SF-36 PCS score (ANOVA trend test; *F* = 2.2; *p* = 0.134).

Figure 1 shows the prevalence of major depression (defined as CES-D ≥ 16) by number of childhood adversities. The burden of depression increased in a linear fashion (from 20% to 61%) with increasing number of childhood adversities (trend test: *p* < 0.001). A dose-response relationship was observed between the number of childhood adversities reported and mental health-related quality of life (ANOVA trend test; *F* = 77.2; *p* < 0.001); that is, participants who reported greater number of adversities had worse lower mean SF-36 MCS score (Figure 2). However, there was no significant linear trend between number of childhood adversities and physical health-related quality of life participants (ANOVA trend test; *F* = 2.2; *p* = 0.134); only individuals with five or more events had significantly lower SF-36 PCS score than those with no adversities.

Results from multivariable linear and logistic regression analyses examining associations between number of childhood adversities and depression are summarized in Table 3. In multivariable logistic regression analysis, the odds of depression increased with increasing number of adversities. Participants who reported four (adjusted odds ratio, AOR = 2.08; 95% CI: 1.16–3.75 *p* = 0.015) or five or more adversities (AOR = 2.10; 95% CI: 1.07–4.14; *p* = 0.032) were twice more likely to experience depression than those who reported no childhood adversity, even after adjusting for demographic, clinical, and psychosocial factors. People who reported three adversities were also 1.6 times more likely to report depression than those with no childhood adversity although the association did not reach statistical significance (AOR = 1.643; 95% CI: 0.96–2.79; *p* = 0.069). We found no significant effect modification by covariate variables in the final model.

We also found independent associations between number of childhood adversities and HRQOL measures. Compared to individuals with no adversity, those with greater number of childhood adversities reported decreasing SF-36 PCS scores after adjusting for demographic, clinical, and psychosocial variables. However, the difference reached statistical significance only among those with five or more adversities (*β* = −3.68; *p* < 0.001) (Table 4). On the other hand, SF-36 MCS decreased in a linear fashion with increasing number of childhood adversities. Relative to those with no adversity, people who reported three adversities (*β* = −3.11; *p* < 0.001), four adversities (*β* = −2.76; *p* = 0.004), and five or more adversities (*β* = −3.27; *p* = 0.003) had significantly worse SF-36 MCS score after adjustment for demographic, clinical, and psychosocial factors (Table 5).

Greater number of child adversities (four or more adversities) was inversely associated with HIV viral load in bivariable logistic regression analyses (Table 6). Participants who reported four adversities (odds ratio, OR = 0.49; 95% CI: 0.31–0.77; *p* = 0.002) or five adversities or greater (OR = 0.50; 95% CI: 0.30–0.83; *p* = 0.007) were significantly less likely to have undetectable viral load in bivariable analyses, but these associations did not reach statistical significance in multivariable regression model. On the other hand, we found no significant statistical association between number of childhood adversities and CD4 count in bivariable or multivariable regression analyses.

## 4. Discussion

We found a high rate of childhood adversities (71%) in this urban sample of people living with HIV who were receiving care. This prevalence was markedly higher than the rate found in the Canadian general population (49%) using the same 7-item survey [27]. This is consistent with other studies suggesting childhood trauma and maltreatment are disproportionately more frequent in people living with HIV [7]. For example, one Canadian study screened 853 patients at an urban HIV clinic and found that 34% reported past or current abuse (physical, sexual, or emotional), and of those who reported abuse, 57% experienced some form of abuse in childhood [14]. Another study among injection drug users living with HIV also reported high prevalence of emotional abuse (51.9%), emotional neglect (36.9%), physical abuse (51.1%), physical neglect (46.8%), and sexual abuse (41.6%) [15].

Prevalence of childhood adversity in our sample varied by age with higher rates in younger than older participants. This is in agreement with findings from the general Canadian population [28]. It is possible that age differences can be explained by recall bias (less forgetting in younger persons) or by reluctance among older persons to report childhood adversity. Race or ethnicity also was a factor, with Indigenous persons reporting the highest rate of childhood adversity, a finding seen in both people with HIV and the general Canadian population [14, 28]. Although there was no significant difference in the overall prevalence of childhood adversity by gender, female and MSM participants reported* physical abuse* more frequently than non-MSM participants. This is consistent with others that reported greater rate of childhood physical and sexual abuse among women and MSM living with HIV [10, 13, 29]. We also found prevalence of* frequent parental substance use* higher among MSM than male non-MSM or female participants.

This study found significant associations between childhood adversities and deleterious health behaviors like cigarette smoking and illicit drug use. Among the health behaviors we surveyed, only hazardous alcohol use was not associated with childhood adversity. This is consistent with Patten et al. [30], who found no association between adversities and alcohol use in the Canadian general population, but contrary to the ACE study in the United States that reported significant association between childhood adversity and increased alcohol consumption [1]. Another study from New Zealand found that childhood maltreatment is associated with lifetime alcohol abuse or dependence, but not with current alcohol use disorder [31]. Although we found some significant association between childhood adversity and HIV viral load among individuals with four adversities or greater, the association did not hold after adjusting for other covariates. Given the strong associations found in other studies [18], further research is needed to understand if this association is mediated or moderated by other variables such as stress and coping.

We observed strong associations between number of childhood adversities and increased odds of depression and worse mental HRQOL. This is consistent with previous research in the general population [2]. It remains unclear, however, what factors mediate and moderate this relationship. Research suggests that adverse experiences in childhood may increase stress reactivity and lead to allostatic overload of the hypothalamic-pituitary-adrenal axis, and that this toxic stress results in neurologic, endocrine, and immunologic changes enduring into adulthood and predisposing to morbidity and mortality [32–34]. As well as physical health outcomes, this physiologic model of impaired stress reactivity has been suggested to mediate increased vulnerability to depression and other mental health outcomes [35]. Further work is needed to understand how this toxic stress impacts HIV, which is already a disease of immune suppression and chronic inflammatory processes.

Our study does have a number of limitations. First, our childhood adversity measure does not contain an item specific to sexual abuse, a critically important adverse childhood experience known to be more prevalent in women, MSM, and people who use drugs [7, 10, 12, 13]. We presume that this form of abuse is captured by one of the seven questions; that is, “Did something happen that scared you so much that you thought about it for years after?” Second, retrospective assessment of childhood experiences may be subject to errors, inconsistencies, and recall bias. This is especially a concern in depressed mood and may increase the likelihood of recalling negative experiences [36]. However, a study that examined the association between childhood adversity and depression using retrospective and prospective measures for childhood adversity found similar results and even found slightly stronger associations with retrospective reporting [31]. Third, some of the questions garnering data on childhood adversity were vague and it was not possible to determine whether they were due to maltreatment or other reasons. For example, we were not able to determine if* lengthy hospital stay* was a result of illness or abuse. Fourth, our study did not assess adulthood traumatic events such as intimate partner violence or physical or emotional abuse. Fifth, the current study uses cross-sectional data and hence we are unable to draw conclusions about the long-term effects of childhood adversities on health outcomes beyond broad associations. Finally, generalizability of our findings is limited as the study sample included individuals attending three urban HIV clinics and was underrepresentative of rural and transgender populations.

Despite these limitations, our study shows a higher prevalence of childhood adversity among people with HIV who are engaged in care than the rates reported in the general population. We have also demonstrated that, even among people living with HIV, childhood adversity rates are higher among those who are younger, ethnic minorities (Indigenous and African, Caribbean, or Black), and those with low socioeconomic status. Further, we have shown that greater number of childhood adversities is independently associated with higher odds of depression and worse physical and mental HRQOL after adjusting for demographic, substance use, and clinical variables. This is the first Canadian study, to our knowledge, to validate the link between childhood adversity and adverse health outcomes using a large diverse sample of people living with HIV that includes Indigenous people, women, MSM, and immigrants. Replication is required with more comprehensive measures that address childhood emotional and sexual abuse, as well as adulthood trauma and violence. As well, further research is needed to understand factors that mediate or moderate the association between childhood adversity and adverse health outcomes in this population. Most of all, this work highlights the need to incorporate screening for childhood adversity and expand clinical services to address adversity and trauma within the context of HIV care. It also shows the need for interventions to prevent childhood adversities and build resilience factors in children and families.

## Figures and Tables

**Table 1:** Sample characteristics and prevalence of childhood adversity.

Characteristics	Total sample		Prevalence childhood adversity^†^	
	*n*	(%)	%	(95% CI)
Whole sample	**1,409**	**(100.0)**	**71.3**	**(68.9, 73.7)**
*Age in years* ^*∗∗*^				
≤30	94	(6.7)	81.9	(74.0, 89.8)
31–40	230	(16.3)	76.1	(70.5, 81.6)
41–50	502	(35.6)	72.1	(68.2, 76.0)
>50	583	(41.4)	67.1	(63.2, 70.9)
*Sex*				
Female	278	(19.7)	73.7	(68.5, 78.9)
Male, MSM	885	(62.8)	71.6	(68.7, 74.6)
Male, non-MSM	246	(17.5)	67.5	(61.6, 73.4)
*Race or ethnicity* ^*∗∗*^				
Indigenous	72	(5.1)	81.8	(73.0, 90.6)
African, Caribbean, or Black	351	(24.9)	76.0	(71.5, 80.5)
White	787	(55.9)	69.9	(66.7, 73.1)
Other, non-White^a^	205	(14.5)	64.9	(58.3, 71.5)
*Immigrant status*				
Born in Canada	792	(56.2)	71.7	(68.6, 74.9)
Immigrant	613	(43.8)	70.8	(67.2, 74.4)
*Marital status*				
Married or living with a partner	186	(13.2)	65.4	(58.5, 72.3)
Single, divorced, or widowed	1,223	(86.8)	72.4	(69.8, 74.9)
*Highest education level* ^*∗*^				
Some high school or less	164	(11.6)	81.1	(75.0, 87.2)
Completed high school	260	(18.5)	73.5	(68.1, 78.9)
Some postsecondary	557	(39.5)	70.0	(66.2, 73.8)
Completed university	428	(30.4)	68.0	(63.6, 72.4)
*Employment status* ^*∗∗*^				
Employed	673	(47.8)	64.9	(61.3, 68.5)
Unemployed-seeking	131	(9.3)	87.8	(82.1, 93.5)
Not in the labour force	275	(19.5)	71.3	(65.9, 76.7)
On disability	330	(23.4)	78.0	(73.5, 82.4)
*Annual personal income* ^*∗∗*^				
<$20,000	598	(42.4)	78.8	(75.5, 82.0)
$20,000–$49,999	464	(32.9)	69.8	(65.6, 74.0)
≥50,000	347	(24.6)	60.5	(55.4, 65.7)
*Hazardous alcohol use* ^b^				
Yes	447	(31.7)	69.8	(65.5, 74.1)
No	962	(68.3)	72.0	(69.2, 74.9)
*Cigarette smoking* ^c*∗∗*^				
Current smoker	407	(28.9)	76.9	(72.8, 81.0)
Former smoker	340	(24.1)	71.5	(66.6, 76.3)
Nonsmoker	662	(47.0)	67.8	(64.3, 71.4)
*Nonmedicinal drug use* ^d*∗*^				
Yes	176	(12.5)	78.4	(72.3, 84.5)
No	1,233	(87.5)	70.3	(67.8, 72.9)
*Years since HIV diagnosis*				
<5	248	(17.6)	72.6	(67.0, 78.2)
5–10	338	(23.9)	74.5	(69.8, 79.2)
11–15	210	(14.9)	68.3	(61.9, 74.6)
16–20	244	(17.3)	70.9	(65.2, 76.6)
>20	369	(26.2)	69.7	(64.9, 74.4)
*Sex with HIV-negative or unknown status partner* ^e*∗*^				
Unknown/missing	279	(19.8)	74.6	(69.4, 79.7)
Not sexually active	551	(39.1)	72.2	(68.5, 76.0)
Yes, with condom	455	(32.3)	66.8	(62.5, 71.2)
Yes, without condom	124	(8.8)	76.6	(69.1, 84.2)
*Recent CD4 count (cells/mm* ^*3*^)^*∗*^				
<200	110	(7.8)	74.5	(66.3, 82.8)
200–499	593	(42.1)	74.4	(70.8, 77.9)
≥500	706	(50.1)	68.3	(64.8, 71.7)
*Recent viral load (copies/mL)* ^*∗*^				
<50	1,127	(80.0)	70.1	(67.4, 72.8)
≥50	282	(20.0)	76.2	(71.2, 81.2)
*On antiretroviral treatment* ^*∗*^				
Yes	1,287	(91.3)	70.3	(75.0, 88.9)
No	122	(8.7)	82.0	(67.8, 72.8)
*Diagnosed with AIDS*				
Yes	613	(43.5)	72.2	(68.6, 75.8)
No	796	(56.5)	70.7	(67.5, 73.8)

^†^One or more childhood adversities.  ^*∗*^Prevalence difference significant at *p *< 0.05 level.  ^*∗∗*^Prevalence difference significant at *p *< 0.001 level.  ^a^Includes people who identified as Asian, Latin American, or mixed races.  ^b^Past 12 months.  ^c^Past 30 days.  ^d^Past six months.  ^e^Past three months. *Note*. Percentages may not add up to 100 due to rounding. *CI*: confidence interval.

**Table 2:** Sociodemographic characteristics and number of childhood adversities.

Characteristics	Number of childhood adversities												*χ* ^2^/*F*	*p* value
	0		1		2		3		4		5–7			
	(*n* = 404)		(*n* = 350)		(*n* = 259)		(*n* = 163)		(*n* = 135)		(*n* = 98)			
	[28.7%]		[24.8%]		[18.4%]		[11.6%]		[9.6%]		[7.0%]			
*Age group*														
≤30	17	(4%)	13	(4%)	24	(9%)	14	(9%)	15	(11%)	11	(11%)	43.7	**<0.001**
31–40	55	(14%)	54	(15%)	47	(18%)	26	(16%)	31	(23%)	17	(17%)		
41–50	140	(35%)	124	(35%)	83	(32%)	61	(37%)	53	(39%)	41	(42%)		
>50	192	(48%)	159	(45%)	105	(41%)	62	(38%)	36	(27%)	29	(30%)		
*Sex*														
Female	73	(18%)	62	(18%)	56	(22%)	36	(22%)	26	(9%)	25	(26%)	10.0	0.445
Male, MSM	251	(62%)	227	(65%)	159	(61%)	105	(64%)	90	(67%)	53	(54%)		
Male, non-MSM	80	(20%)	61	(17%)	44	(17%)	22	(13%)	19	(14%)	20	(20%)		
*Race or ethnicity*														
Indigenous	14	(3%)	18	(5%)	15	(6%)	9	(6%)	9	(7%)	7	(7%)	18.1	0.258
African/Black/Caribbean	83	(21%)	88	(25%)	73	(28%)	44	(27%)	42	(31%)	21	(21%)		
Other, non-White	72	(18%)	49	(14%)	31	(12%)	25	(15%)	16	(12%)	12	(12%)		
White	235	(58%)	195	(56%)	140	(54%)	85	(52%)	68	(50%)	58	(59%)		
*Born in Canada*														
No	178	(44%)	163	(47%)	112	(43%)	63	(39%)	65	(48%)	32	(33%)	9.2	0.103
Yes	224	(55%)	186	(53%)	146	(56%)	100	(61%)	70	(52%)	66	(67%)		
*Marital status*														
Married/committed relationship	64	(16%)	42	(12%)	32	(12%)	18	(11%)	12	(9%)	18	(18%)	8.7	0.121
Single/divorced/widowed	337	(83%)	307	(88%)	227	(88%)	145	(89%)	123	(91%)	79	(81%)		
*Highest education level*														
Some high school or less	31	(8%)	36	(10%)	33	(13%)	22	(13%)	19	(14%)	23	(23%)	43.8	**<0.001**
Completed high school	69	(17%)	72	(21%)	41	(16%)	24	(15%)	33	(24%)	21	(21%)		
College/some university	167	(41%)	124	(35%)	100	(39%)	78	(48%)	55	(41%)	33	(34%)		
Completed university	137	(34%)	118	(34%)	85	(33%)	39	(24%)	28	(21%)	21	(21%)		
*Employment status*														
Employed	236	(58%)	171	(49%)	108	(16%)	69	(42%)	54	(40%)	35	(36%)	54.9	**<0.001**
Unemployed and seeking	16	(4%)	29	(8%)	33	(25%)	26	(16%)	15	(11%)	12	(12%)		
Not in labour force	79	(20%)	68	(19%)	55	(20%)	24	(15%)	28	(21%)	21	(21%)		
On disability	73	(18%)	82	(23%)	63	(19%)	44	(27%)	38	(28%)	30	(31%)		
*Annual personal income*													69.6	**<0.001**
<$20,000	127	(31%)	136	(39%)	120	(46%)	75	(46%)	83	(61%)	57	(58%)		
$20,000–$49,999	140	(35%)	118	(34%)	90	(35%)	49	(30%)	37	(27%)	30	(31%)		
≥$50,000	137	(34%)	96	(27%)	49	(19%)	39	(24%)	15	(11%)	11	(11%)		
*Hazardous alcohol use* ^a^														
Yes	135	(33%)	118	(34%)	73	(28%)	48	(29%)	41	(30%)	32	(33%)	3.2	0.667
No	269	(67%)	232	(66%)	186	(72%)	115	(71%)	94	(70%)	66	(67%)		
*Cigarette smoking* ^b^														
Smoker	94	(23%)	95	(27%)	77	(30%)	55	(34%)	46	(34%)	40	(41%)	22.7	**0.004** ^c^
Nonsmoker	310	(77%)	255	(73%)	182	(70%)	108	(66%)	89	(66%)	58	(59%)		
*Nonmedicinal drug use* ^d^														
Yes	38	(9%)	50	(14%)	32	(12%)	15	(9%)	20	(15%)	21	(21%)	14.0	**0.016** ^c^
No	366	(91%)	300	(86%)	227	(88%)	148	(91%)	115	(85%)	77	(79%)		
*Sex with HIV-negative/unknown partner* ^f^														
Unknown/missing	71	(18%)	71	(20%)	41	(16%)	35	(21%)	33	(24%)	28	(29%)	12.9	0.230
Not sexually active	153	(38%)	146	(42%)	114	(44%)	57	(35%)	48	(36%)	33	(34%)		
Had sex with condom	151	(37%)	104	(30%)	78	(30%)	56	(34%)	42	(31%)	24	(24%)		
Had sex without condom	29	(7%)	29	(8%)	26	(10%)	15	(9%)	12	(9%)	13	(13%)		
*On antiretroviral treatment*														
Yes	382	(95%)	315	(90%)	240	(93%)	148	(91%)	120	(89%)	82	(84%)	15.0	**0.010** ^c^
No	22	(5%)	35	(10%)	19	(7%)	15	(9%)	15	(11%)	16	(16%)		
*Recent CD4 count (cells/mm* ^*3*^)														
<200	28	(7%)	33	(9%)	21	(8%)	11	(7%)	7	(5%)	10	(10%)	12.6	0.245
200–499	152	(38%)	160	(46%)	116	(45%)	65	(40%)	59	(44%)	41	(42%)		
≥500	224	(55%)	157	(45%)	122	(47%)	87	(53%)	69	(51%)	47	(48%)		
*Recent viral load (copies/mL)* ^*∗*^														
<50	337	(83%)	278	(79%)	207	(80%)	139	(85%)	96	(71%)	70	(71%)	17.1	**0.005** ^c^
≥50	67	(17%)	72	(21%)	52	(20%)	24	(15%)	39	(29%)	28	(29%)		
*Diagnosed with AIDS*														
Yes	167	(41%)	164	(47%)	119	(46%)	66	(40%)	52	(39%)	45	(46%)	5.1	0.399
No	237	(59%)	186	(53%)	140	(54%)	97	(60%)	83	(61%)	53	(54%)		

^a^Past 12 months.  ^b^Past 30 days.  ^c^Significant linear trend, *p* < 0.05.  ^d^Past six months.  ^e^Includes blood transfusion and mother-to-child transmission.  ^f^Past 3 months.

**Table 3:** Childhood adversities as predictors of depression (CES-D ≥ 16): results from bivariable and multivariable logistic regression models.

Predictor variable	Bivariable regression model		Multivariable regression model	
	OR (95% CI)	*p*	AOR (95% CI)	*p*
*Number of childhood adversity*				
Five to seven	6.39 (3.98, 10.28)	**<0.001**	2.10 (1.07, 4.14)	**0.032**
Four	4.36 (2.87, 6.62)	**<0.001**	2.08 (1.16, 3.75)	**0.015**
Three	3.28 (2.22, 4.87)	**<0.001**	1.64 (0.96, 2.79)	0.069
Two	1.91 (1.34, 2.73)	**<0.001**	1.03 (0.64, 1.66)	0.902
One	1.67 (1.19, 2.33)	**0.003**	1.14 (0.73, 1.79)	0.568
None (ref)	1.00		1.00	
*Age in years*				
>50	0.67 (0.43, 1.05)	0.081	0.88 (0.42, 1.85)	0.745
41–50	0.86 (0.55, 1.36)	0.523	1.06 (0.53, 2.15)	0.862
31–40	0.96 (0.59, 1.58)	0.879	0.80 (0.39, 1.64)	0.541
≤30 (ref)	1.00		1.00	-
*Sex/gender*				
Female	1.46 (1.02, 2.08)	**0.036**	1.80 (1.08, 3.02)	**0.025**
Male, MSM	0.80 (0.59, 1.08)	0.137	1.33 (0.84, 2.10)	0.227
Male, non-MSM (ref)	1.00		1.00	
*Race/ethnicity *				
Indigenous	1.65 (1.02, 2.67)	**0.040**	0.70 (0.36, 1.43)	0.324
African/Caribbean/Black	1.52 (1.17, 1.98)	**0.002**	0.40 (0.24, 0.67)	**0.001**
Other, non-White	1.05 (0.76, 1.47)	0.754	0.67 (0.42, 1.07)	0.090
White (ref)			1.00	-
*Education*				
Less than HS completion	3.96 (2.70, 5.80)	**<0.001**	1.48 (0.85, 2.60)	0.168
Completed high school	1.92 (1.36, 2.70)	**<0.001**	1.16 (0.71, 1.89)	0.553
Some postsecondary	1.87 (1.41, 2.50)	**<0.001**	1.53 (1.02, 2.29)	**0.042**
Completed university (ref)	1.00		1.00	
*Born in Canada*				
Yes	0.88 (0.70, 1.10)	0.251	-	-
No (ref)	1.00		-	-
*Currently employed*				
Yes	0.39 (0.31, 0.50)	**<0.001**	0.72 (0.51, 1.00)	0.053
No (ref)	1.00		1.00	
*Hazardous alcohol use *				
Yes	0.73 (0.58, 0.94)	**0.013**	0.88 (0.61, 1.25)	0.462
No (ref)	1.00		1.00	
*Cigarette smoking*				
Current smoker	1.60 (1.23, 2.07)	**<0.001**	0.84 (0.56, 1.26)	0.391
Former smoker	1.05 (0.79, 1.39)	0.751	0.88 (0.58, 1.34)	0.543
Never smoker (ref)	1.00		1.00	
*Nonmedicinal drug use*				
Yes	1.39 (1.00, 1.92)	**0.049**	1.03 (0.63, 1.69)	0.905
No (ref)	1.00		1.00	
*Duration of HIV infection *				
Years (per 10 years)	0.88 (0.77, 1.01)	0.080	1.00 (0.79, 1.27)	0.995
*Diagnosed with AIDS*				
Yes	0.92 (0.73, 1.15)	0.448	-	-
No (ref)	1.00		-	-
*Recent CD4 count (cells/mm* ^*3*^)				
≥500	0.68 (0.45, 1.03)	0.066	0.83 (0.44, 1.55)	0.558
200–499	0.72 (0.47, 1.09)	0.120	0.84 (0.45, 1.56)	0.577
<200 (ref)	1.00		1.00	
*HIV viral load (copies/mL)*				
≥50	1.37 (1.05, 1.80)	**0.021**	0.84 (0.51, 1.39)	0.499
<50 (ref)	1.00		1.00	
*On antiretroviral treatment *				
Yes	0.69 (0.47, 1.01)	0.058	1.06 (0.54, 2.09)	0.864
No (ref)	1.00		1.00	

Bold face indicates statistical significance (*p* < 0.05). *Note*. Variables with *p* < 0.10 in bivariable regression models were included in the multivariable regression model. The final multivariable model is further adjusted for mastery, social support, coping, and stress.

**Table 4:** Childhood adversities as predictors of SF-36 physical component summary (PCS) score: results from bivariable and multivariable linear regression models.

Predictor variable	Bivariable regression model			Multivariable regression model		
	*β*	(95% CI)	*p*	*β*	(95% CI)	*p*
*Number of childhood adversity*						
Five to seven	−4.46	(−6.17, −2.74)	**<0.001**	−3.68	(−5.49, −1.88)	**<0.001**
Four	−0.71	(−2.21,0.79)	0.354	−0.93	(−2.52,0.65)	0.248
Three	0.69	(−0.69,2.06)	0.330	0.61	(−0.85,2.07)	0.413
Two	−0.38	(−1.51,0.76)	0.518	−0.40	(−1.64,0.84)	0.524
One	0.47	(−0.55,1.49)	0.364	−0.03	(−1.16,1.09)	0.953
None (ref)	0	-	-	0	-	-
*Age in years*						
>50	−2.36	(−3.25, −1.48)	**<0.001**	−3.59	(−5.56, −1.62)	**<0.001**
41–50	0.27	(−0.66,1.19)	0.572	−2.42	(−4.27, −0.57)	**<0.001**
31–40	1.93	(0.74,3.12)	**0.002**	−1.17	(−3.08,0.74)	0.230
≤30 (ref)	0	-	-	0	-	-
*Sex/gender*						
Female	−0.58	(−1.69,0.53)	0.303	−0.39	(−1.78,1.00)	0.584
Male, MSM	1.08	(0.17,1.99)	**0.020**	1.14	(−0.07,2.34)	0.064
Male, non-MSM (ref)	0	-	-	0	-	-
*Race/ethnicity *						
Indigenous	−2.17	(−4.11, −0.23)	**0.028**	−0.63	(−2.48,1.22)	0.503
African/Caribbean/Black	1.00	(−0.03,2.02)	0.057	0.04	(−1.55,1.63)	0.962
Other, non-White	0.23	(−1.02,1.48)	0.717	−0.95	(−2.40,0.50)	0.198
White (ref)	0	-	-	0	-	-
*Education*						
Less than HS completion	−3.24	(−4.61, −1.88)	**<0.001**	−1.53	(−3.05,0.00)	0.050
Completed high school	−1.38	(−2.51, −0.24)	**0.017**	−1.17	(−2.42,0.09)	0.070
Some postsecondary	0.34	(−0.57,1.24)	0.465	−0.82	(−1.84,0.20)	0.113
Completed University (ref)	0	-	-	0	-	-
*Born in Canada*						
Yes	−1.34	(−2.22, −0.45)	**0.003**	−1.33	(−2.58, −0.09)	**0.036**
No (ref)	0	-	-	0	-	-
*Currently employed*						
Yes	4.75	(3.91,5.60)	**<0.001**	3.34	(2.45,4.23)	**<0.001**
No (ref)	0	-	-	0	-	-
*Hazardous alcohol use *						
Yes	1.45	(0.50,2.39)	**0.003**	1.03	(0.11,1.94)	**0.029**
No (ref)	0	-	-	0	-	-
*Cigarette smoking*						
Current smoker	−1.85	(−2.82, −0.88)	**<0.001**	−1.24	(−2.31, −0.17)	**0.024**
Former smoker	−0.67	(−1.70,0.36)	0.205	−0.83	(−1.91,0.25)	0.131
Never smoker (ref)	0	-	-	0	-	-
*Nonmedicinal drug use*						
Yes	−0.28	(−1.61,1.06)	0.683	-	-	-
No (ref)	0	-	-	-	-	-
*Duration of HIV infection *						
Years (per 10 years)	−1.81	(−2.35, −1.27)	**<0.001**	−0.68	(−1.31, −0.04)	**0.036**
*Diagnosed with AIDS*						
Yes	−1.96	(−2.84, −1.07)	**<0.001**	−0.83	(−1.69,0.03)	0.057
No (ref)	0	-	-	0	-	-
*Recent CD4 count (cells/mm* ^*3*^)						
≥500	1.19	(0.31,2.07)	**0.008**	1.41	(−0.24,3.06)	0.093
200–499	−0.40	(−1.29,0.49)	0.382	1.08	(−0.58,2.73)	0.202
<200 (ref)	0	-	-	0	-	-
*HIV viral load (copies/mL)*						
≥50	−1.17	(−2.28, −0.07)	**0.037**	−0.98	(−2.07,0.11)	0.079
<50 (ref)	0	-	-	0	-	-

Bold face indicates statistical significance (*p* < 0.05). *Note*. Variables with *p* < 0.10 in bivariable regression models were included in the multivariable regression model. The final multivariable model is further adjusted for mastery, social support, coping, and stress.

**Table 5:** Childhood adversities as predictors of SF-36 mental component summary (MCS) score: results from bivariable multivariable linear regression models.

Predictor variable	Bivariable linear regression model			Multivariable linear regression model		
	*β*	(95% CI)	*p*	*β*	(95% CI)	*p*
*Number of childhood adversity*						
Five to seven	−8.50	(−11.01, −6.00)	**<0.001**	−3.27	(−5.39, −1.14)	**0.003**
Four	−5.28	(−7.46, −3.09)	**<0.001**	−2.76	(−4.62, −0.89)	**0.004**
Three	−4.32	(−6.34, −2.31)	**<0.001**	−3.11	(−4.83, −1.40)	**<0.001**
Two	−0.37	(−2.05,1.30)	0.662	−1.40	(−2.86,0.05)	0.059
One	1.69	(0.19,3.18)	**0.027**	−0.67	(−2.00,0.65)	0.319
None (ref)	0	-	-	0	-	-
*Age in years*						
>50	3.03	(1.72,4.33)	**<0.001**	2.55	(0.23,4.87)	**0.031**
41–50	−0.52	(−1.87,0.84)	0.454	1.38	(−0.79,3.55)	0.213
31–40	−2.87	(−4.62, −1.12)	**0.001**	0.66	(−1.60,2.91)	0.567
≤30 (ref)	0	-	-	0	-	-
*Sex/gender*						
Female	−2.50	(−4.12, −0.88)	**0.003**	−1.53	(−3.16,0.11)	0.067
Male, MSM	1.65	(0.31,2.98)	**0.016**	−0.71	(−2.13,0.71)	0.325
Male, non-MSM (ref)	0	-	-	0	-	-
*Race/ethnicity *						
Indigenous	−2.82	(−5.66,0.03)	0.053	0.80	(−1.38,2.98)	0.473
African/Caribbean/Black	−0.66	(−2.16,0.85)	0.390	4.67	(3.16,6.18)	**<0.001**
Other, non-White	−0.13	(−1.97,1.71)	0.889	1.50	(0.04,2.95)	**0.043**
White (ref)	0	-	-	0	-	-
*Education*						
Less than HS completion	−3.53	(−5.54, −1.52)	**0.001**	1.91	(0.11,3.71)	**0.038**
Completed high school	−0.41	(−2.08,1.26)	0.631	1.03	(−0.46,2.51)	0.175
Some postsecondary	−0.47	(−1.80,0.85)	0.485	−0.13	(−1.32,1.07)	0.836
Completed university (ref)	0	-	-	0	-	-
*Born in Canada*						
Yes	−0.24	(−1.55,1.07)	0.719	-	-	-
No (ref)	0	-	-	-	-	-
*Currently employed*						
Yes	4.48	(3.20,5.75)	**<0.001**	0.91	(−0.14,1.95)	0.089
No (ref)	0	-	-	0	-	-
*Hazardous alcohol use *						
Yes	0.98	(−0.41,2.38)	0.165	-	-	-
No (ref)	0	-	-	-	-	-
*Cigarette smoking*						
Current smoker	−2.74	(−4.17, −1.32)	**<0.001**	0.99	(−0.26,2.23)	0.122
Former smoker	0.94	(−0.58,2.45)	0.225	0.41	(−0.86,1.68)	0.523
Never smoker (ref)	0	-	-	0	-	-
*Nonmedicinal drug use*						
Yes	−3.62	(−5.57, −1.66)	**<0.001**	−0.41	(−1.95,1.13)	0.603
No (ref)	-	-	-	0	-	-
*Duration of HIV infection *						
Years (per 10 years)	1.52	(0.72,2.32)	**<0.001**	0.23	(−0.51,0.97)	0.541
*Diagnosed with AIDS*						
Yes	0.86	(−0.46,2.17)	0.201	-	-	-
No (ref)	0	-	-	-	-	-
*Recent CD4 count (cells/mm* ^*3*^)						
≥500	0.71	(−0.59,2.00)	0.283	0.05	(−1.91,2.01)	0.957
200–499	−0.05	(−1.36,1.26)	0.939	0.08	(−1.88,2.04)	0.935
<200 (ref)	0	-	-	0	-	-
*HIV viral load (copies/mL)*						
≥50	−3.38	(−4.99, −1.77)	**<0.001**	−0.72	(−1.96,0.51)	0.251
<50 (ref)	0	-	-	0	-	-

Bold face indicates statistical significance (*p* < 0.05). *Note*. Variables with *p* < 0.10 in bivariable regression models were included in the multivariable regression model. The final multivariable model is further adjusted for mastery, social support, coping, and stress.

**Table 6:** Childhood adversities as predictors of undetectable viral load (<50 copies/mL) and CD4 count (≥200 cells/mm^3^): results from bivariable and multivariable logistic regression models.

Predictor variable	Undetectable viral load (<50 copies/mL)				CD4 count (≥200 cells/mm^3^)			
	Bivariable regression model		Multivariable regression model		Bivariable regression model		Multivariable regression model	
	OR (95% CI)	*p*	AOR (95% CI)	*p*	OR (95% CI)	*p*	AOR (95% CI)	*p*
*Childhood adversity (number)*								
Five to seven	0.50 (0.30, 0.83)	**0.007**	0.83 (0.47, 1.46)	0.518	0.66 (0.31, 1.40)	0.275	0.98 (0.43, 2.24)	0.966
Four	0.49 (0.31, 0.77)	**0.002**	0.69 (0.42, 1.13)	0.142	1.36 (0.58, 3.19)	0.478	1.91 (0.78, 4.67)	0.156
Three	1.15 (0.69, 1.91)	0.585	1.44 (0.84, 2.47)	0.184	1.03 (0.50, 2.12)	0.938	1.32 (0.62, 2.81)	0.473
Two	0.79 (0.53, 1.18)	0.254	0.92 (0.60, 1.41)	0.706	0.84 (0.47, 1.52)	0.572	0.98 (0.53, 1.81)	0.955
One	0.77 (0.53, 1.11)	0.160	0.85 (0.57, 1.24)	0.395	0.72 (0.42, 1.21)	0.211	0.78 (0.45, 1.33)	0.363
None (ref)	1.00		1.00	-	1.00		1.00	
*Age in years*								
>50	3.84 (2.35, 6.27)	**<0.001**	2.20 (1.21, 4.00)	**0.010**	1.01 (0.41, 2.45)	0.989	1.54 (0.55, 4.33)	0.414
41–50	1.93 (1.20, 3.10)	**0.007**	1.38 (0.80, 2.35)	0.244	0.60 (0.25, 1.45)	0.258	0.86 (0.33, 2.23)	0.750
31–40	1.32 (0.79, 2.19)	0.291	1.13 (0.66, 1.96)	0.654	0.91 (0.35, 2.41)	0.852	1.11 (0.40, 3.03)	0.845
≤30 (ref)	1.00			-	1.00		1.00	
*Sex/gender*								
Female	1.36 (0.91, 2.03)	0.133	1.39 (0.90, 2.15)	0.139	1.59 (0.87, 2.91)	0.133	1.57 (0.82, 2.99)	0.173
Male, MSM	1.76 (1.27, 2.45)	**0.001**	1.57 (1.08, 2.29)	**0.020**	1.61 (1.00, 2.59)	0.050	1.36 (0.80, 2.32)	0.253
Male, non-MSM (ref)	1.00		1.00	-	1.00		1.00	
*Race/ethnicity *								
Indigenous	0.72 (0.41, 1.25)	0.241	1.07 (0.59, 1.95)	0.824	0.76 (0.33, 1.73)	0.509	1.00 (0.43, 2.35)	0.999
African/Caribbean/Black	0.78 (0.57, 1.06)	0.113	1.27 (0.83, 1.94)	0.267	0.86 (0.54, 1.38)	0.534	0.97 (0.52, 1.80)	0.920
Other, non-White	0.71 (0.49, 1.04)	0.076	0.86 (0.57, 1.28)	0.449	0.70 (0.41, 1.20)	0.194	0.76 (0.43, 1.34)	0.344
White (ref)	1.00		1.00		1.00		1.00	
*Education*								
Less than HS completion	0.60 (0.39, 0.92)	**0.020**	0.95 (0.57, 1.56)	0.827	0.50 (0.27, 0.95)	**0.034**	0.68 (0.34, 1.37)	0.279
Completed high school	0.60 (0.41, 0.87)	**0.008**	0.72 (0.48, 1.10)	0.128	0.49 (0.28, 0.86)	**0.013**	0.63 (0.35, 1.13)	0.123
Some postsecondary	0.86 (0.61, 1.19)	0.359	0.98 (0.69, 1.40)	0.925	0.85 (0.50, 1.43)	0.533	0.94 (0.55, 1.62)	0.825
Completed university (ref)	1.00		1.00		1.00		1.00	
*Born in Canada*								
Yes	1.14 (0.88, 1.49)	0.314	-	-	1.08 (0.73, 1.59)	0.714	-	-
No (ref)	1.00		-	-	1.00		-	-
*Currently employed*								
Yes	1.25 (0.96, 1.63)	0.096	1.25 (0.92, 1.69)	0.154	1.46 (0.98, 2.18)	0.061	1.28 (0.83, 1.99)	0.268
No (ref)	1.00		1.00		1.00		1.00	
*Hazardous alcohol use *								
Yes	0.95 (0.72, 1.26)	0.717	-	-	1.04 (0.68, 1.59)	0.848	-	-
No (ref)	1.00		-	-	1.00		-	-
*Cigarette smoking*								
Current smoker	0.57 (0.43, 0.77)	**<0.001**	0.69 (0.49, 0.97)	**0.035**	0.63 (0.41, 0.98)	**0.042**	0.77 (0.47, 1.26)	0.295
Former smoker	1.20 (0.84, 1.72)	0.313	1.08 (0.73, 1.60)	0.692	1.01 (0.60, 1.69)	0.984	1.03 (0.59, 1.80)	0.919
Never smoker (ref)	1.00		1.00		1.00		1.00	
*Nonmedicinal drug use*								
Yes	0.53 (0.37, 0.75)	**<0.001**	0.77 (0.51, 1.14)	0.193	1.07 (0.59, 1.95)	0.824	1.25 (0.65, 2.42)	0.502
No (ref)	1.00		1.00		1.00		1.00	
*Duration of HIV infection *								
Years (per 10 years)	1.71 (1.44, 2.03)	**<0.001**	1.38 (1.11, 1.71)	**0.004**	0.85 (0.67, 1.08)	0.177	0.75 (0.55, 1.02)	0.067

*AOR*: adjusted odds ratio; *CI*: confidence interval. Bold face indicates statistical significance (*p* < 0.05). *Note*. Variables with *p* < 0.10 in bivariable regression models were included in multivariable regression models. The final multivariable models are further adjusted for mastery, social support, coping, and stress.

## Supplementary Materials

Supplementary Materials Supplemental Table 1: prevalence of individual childhood adverse events by sociodemographic characteristics (*N* = 1,409).
