# Supplementary material for: Childhood Adversities and Physical and Mental Health Outcomes in Adults Living with HIV: Findings from the Ontario HIV Treatment Network Cohort Study
Source: AIDS Res Treat. 2018 Mar 1;2018:2187232. doi: 10.1155/2018/2187232 (PMC5852860; doi:10.1155/2018/2187232)
Supplement: Supplementary Materials — Supplemental Table 1: prevalence of individual childhood adverse events by sociodemographic characteristics (N = 1,409). [file 2187232.f1.docx]

**Supplemental table 1.**  Prevalence of individual childhood adverse events by sociodemographic characteristics (N=1,409)

| **Demographic characteristics** | **Prevalence of individual childhood adverse event (%, 95% confidence interval)** | | | | | | | | | | | | | | | | | | | | | |
| --- | --- | --- | --- | --- | --- | --- | --- | --- | --- | --- | --- | --- | --- | --- | --- | --- | --- | --- | --- | --- | --- | --- |
|  | **Scary traumatic event** | | | **Physical abuse** | | | **Frequent parental substance use** | | | **Parental divorce** | | | **Lengthy**  **hospital stay** | | | **Lengthy parental**  **unemployment** | | | **Sent away from home** | | | |
| Whole sample |  |  |  |  |  |  |  |  |  |  |  |  |  |  |  |  |  |  |  |  |  |  |
|  | 43.9 | (41.3, | 46.5) | 27.8 | (25.4 | 30.2) | 26.3 | (24.1 | 28.7) | 22.9 | (20.8, | 25.2) | 21.7 | (19.5, | 23.9) | 19.0 | (16.9, | 21.1) | 10.9 | (29.3, | 12.6) |  |
| **Age group** |  |  |  |  |  |  |  |  |  |  |  |  |  |  |  |  |  |  |  |  |  |  |
| ≤30 | 61.7 | (51.7, | 71.7) | 34.0 | (24.3, | 43.8) | 29.8 | (20.4, | 39.2) | 27.7 | (18.4, | 36.9) | 20.2 | (11.9, | 28.5) | 35.1 | (25.3, | 44.9) | 26.6 | (17.5, | 35.7) |  |
| 31-40 | 50.0 | (43.5, | 56.5) | 32.2 | (26.1, | 38.3) | 19.6 | (14.4, | 24.7) | 30.4 | (24.4, | 36.4) | 17.8 | (12.8, | 22.8) | 26.5 | (20.8, | 32.3) | 15.7 | (10.9, | 20.4) |  |
| 41-50 | 44.6 | (40.3, | 49.0) | 28.5 | (24.5, | 32.4) | 28.9 | (24.9, | 32.9) | 25.9 | (22.1, | 29.7) | 24.1 | (20.3, | 27.9) | 18.1 | (14.7, | 21.5) | 10.6 | (7.9, | 13.3) |  |
| >50 | 37.9 | (34.0, | 41.9) | 24.4 | (20.9, | 27.9) | 26.2 | (22.7, | 29.8) | 16.6 | (13.6, | 19.7) | 21.3 | (17.9, | 24.6) | 14.1 | (11.2, | 16.9) | 6.7 | (4.7, | 8.7) |  |
|  | ***p<0.001*** | | | *p=0.055* | | | *p=0.053* | | | ***p<0.001*** | | | *p=0.269* | | | ***p<0.001*** | | | ***p<0.001*** | | | |
| **Sex/gender** |  |  |  |  |  |  |  |  |  |  |  |  |  |  |  |  |  |  |  |  |  |  |
| Female | 47.5 | (41.6, | 53.4) | 34.5 | (28.9, | 40.2) | 22.7 | (17.7, | 27.6) | 25.2 | (20.0, | 30.3) | 21.2 | (16.4, | 26.1) | 24.5 | (19.4, | 29.5) | 11.5 | (7.7, | 15.3) |  |
| Male, MSM | 43.4 | (40.1, | 46.7) | 28.2 | (25.3, | 31.2) | 29.0 | (26.0, | 32.0) | 22.3 | (19.5, | 25.0) | 20.5 | (17.8, | 23.1) | 17.4 | (14.9, | 19.9) | 9.2 | (7.2, | 11.1) |  |
| Male, other | 41.5 | (35.3, | 47.7) | 18.3 | (13.4, | 23.2) | 20.7 | (15.6, | 25.8) | 22.8 | (17.5, | 28.0) | 26.4 | (20.9, | 32.0) | 18.3 | (13.4, | 23.2) | 16.3 | (11.6, | 20.9) |  |
|  | *p=0.344* | | | ***p<0.001*** | | | ***p=0.009*** | | | *p=0.599* | | | *p=0.130* | | | ***p=0.031*** | | | ***p=0.006*** | | | |
| **Race/ethnicity** |  |  |  |  |  |  |  |  |  |  |  |  |  |  |  |  |  |  |  |  |  |  |
| Indigenous | 40.3 | (29.1, | 51.5) | 41.6 | (30.3, | 52.8) | 46.8 | (35.4, | 58.2) | 24.7 | (14.8, | 34.5) | 27.3 | (17.1, | 37.4) | 22.1 | (12.6, | 31.6) | 10.4 | (3.4, | 17.4) |  |
| ACB  Black | 51.7 | (46.4, | 57.0) | 30.9 | (26.0, | 35.8) | 15.6 | (11.8, | 19.4) | 26.9 | (22.2, | 31.6) | 20.5 | (16.2, | 24.8) | 23.7 | (19.2, | 28.2) | 12.7 | (9.2, | 16.2) |  |
| White | 41.5 | (38.0, | 44.9) | 25.7 | (22.7, | 28.8) | 30.7 | (27.5, | 34.0) | 21.3 | (18.4, | 24.1) | 22.5 | (19.6, | 25.5) | 16.4 | (13.8, | 19.0) | 10.4 | (8.2, | 12.5) |  |
| Other | 41.0 | (34.2, | 47.8) | 24.9 | (18.9, | 30.8) | 20.0 | (14.5, | 25.5) | 22.0 | (16.2, | 27.7) | 18.0 | (12.7, | 23.4) | 19.5 | (14.0, | 25.0) | 9.8 | (5.7, | 13.9) |  |
|  | ***p=0.009*** | | | ***p=0.009*** | | | ***p<0.001*** | | | *p=0.209* | | | *p=0.305* | | | ***p=0.030*** | | | *p=0.637* | | | |
| **Born in Canada** |  |  |  |  |  |  |  |  |  |  |  |  |  |  |  |  |  |  |  |  |  |  |
| No | 45.1 | (41.1, | 49.0) | 27.2 | (23.7, | 30.8) | 17.2 | (14.2, | 20.2) | 22.5 | (19.2, | 25.8) | 19.9 | (16.8, | 23.1) | 21.2 | (18.0, | 24.5) | 10.7 | (8.3, | 13.1) |  |
| Yes | 42.9 | (39.5, | 46.4) | 28.2 | (25.0, | 31.3) | 33.5 | (30.2, | 36.8) | 23.2 | (20.3, | 26.2) | 23.0 | (20.0, | 25.9) | 17.2 | (14.5, | 19.8) | 11.0 | (8.8, | 13.2) |  |
|  | *p=0.425* | | | *p=0.699* | | | ***p<0.001*** | | | *p=0.755* | | | *p=0.169* | | | *p=0.054* | | | *p=0.863* | | | |
| **MSM**, men who have sex with men; **ACB**, African, Caribbean, or Black; **CI**, confidence interval  **Note:** P-values are from two-tailed chi-square independence tests. Bold face indicates statistical significance (*p*<0.05). | | | | | | | | | | | | | | | | | | | | | | |
